# Use of Morphometric Mapping to Characterise Symptomatic Chiari-Like Malformation, Secondary Syringomyelia and Associated Brachycephaly in the Cavalier King Charles Spaniel

**DOI:** 10.1371/journal.pone.0170315

**Published:** 2017-01-25

**Authors:** Susan P. Knowler, Chloe Cross, Sandra Griffiths, Angus K. McFadyen, Jelena Jovanovik, Anna Tauro, Zoha Kibar, Colin J. Driver, Roberto M. La Ragione, Clare Rusbridge

**Affiliations:** 1 School of Veterinary Medicine, Faculty of Health & Medical Sciences, University of Surrey, Guildford, Surrey, United Kingdom; 2 Stone Lion Veterinary Hospital, Wimbledon,United Kingdom; 3 akm-stats, Glasgow, Scotland, United Kingdom; 4 Fitzpatrick Referrals, Godalming, Surrey, United Kingdom; 5 CHU Sainte Justine Research Center and University of Montreal, Quebec, Canada; University of Sydney Faculty of Veterinary Science, AUSTRALIA

## Abstract

**Objectives:**

To characterise the symptomatic phenotype of Chiari-like malformation (CM), secondary syringomyelia (SM) and brachycephaly in the Cavalier King Charles Spaniel using morphometric measurements on mid-sagittal Magnetic Resonance images (MRI) of the brain and craniocervical junction.

**Methods:**

This retrospective study, based on a previous quantitative analysis in the Griffon Bruxellois (GB), used 24 measurements taken on 130 T1-weighted MRI of hindbrain and cervical region. Associated brachycephaly was estimated using 26 measurements, including rostral forebrain flattening and olfactory lobe rotation, on 72 T2-weighted MRI of the whole brain. Both study cohorts were divided into three groups; Control, CM pain and SM and their morphometries compared with each other.

**Results:**

Fourteen significant traits were identified in the hindbrain study and nine traits in the whole brain study, six of which were similar to the GB and suggest a common aetiology. The Control cohort had the most elliptical brain (p = 0.010), least olfactory bulb rotation (p = 0.003) and a protective angle (p = 0.004) compared to the other groups. The CM pain cohort had the greatest rostral forebrain flattening (p = 0.007), shortest basioccipital (p = 0.019), but a greater distance between the atlas and basioccipital (p = 0.002) which was protective for SM. The SM cohort had two conformation anomalies depending on the severity of craniocervical junction incongruities; i) the proximity of the dens (p <0.001) ii) increased airorhynchy with a smaller, more ventrally rotated olfactory bulb (p <0.001). Both generated ‘concertina’ flexures of the brain and craniocervical junction.

**Conclusion:**

Morphometric mapping provides a diagnostic tool for quantifying symptomatic CM, secondary SM and their relationship with brachycephaly. It is hypothesized that CM pain is associated with increased brachycephaly and SM can result from different combinations of abnormalities of the forebrain, caudal fossa and craniocervical junction which compromise the neural parenchyma and impede cerebrospinal fluid flow.

## Introduction

Syringomyelia (SM) secondary to Chiari-like Malformation (CM) in the Cavalier King Charles Spaniel (CKCS) has been well documented over the last decade [1–3]. There is a high prevalence of SM in the breed for which CM is ubiquitous [4,5]. Canine CM is generally considered analogous to human Chiari-1 malformation and defined on the basis of the cerebellum being compacted into or herniated through the foramen magnum [6,7]. Aberrations of skull and brain morphology in CM can result in fluid cavitation of the spinal cord (syrinx or syringes) and this can develop progressively over time [8,9]. Reduced volume of the caudal fossa [10,11], cerebellar volume and herniation [12,13] medullary elevation (kinking) [13,14], jugular foramina [15,16], venous sinus volume [17] and atlanto-occipital overlapping [18,19] have all been shown to increase the risk of syringomyelia [20,21]. Evidence suggests that the early closure of skull bone sutures (craniosynostosis) in CKCS [9,22,23] and GB [15,24] reduces the size of the caudal fossa, alters the neuro-parenchymal morphology and disrupts cerebrospinal fluid (CSF) dynamics [20]. Brachycephaly is a risk factor for SM and it is not fully understood how this and craniocervical junction abnormalities such as atlanto-occipital overlapping and medullary position interact with each other to contribute to the severity of CM and SM in the breed. Variable clinical signs are characterised by pain and / or sensory and motor neurological deficits depending on the site and extent of spinal cord damage [8,25,26]. However some dogs with CM alone exhibit behavioural signs of pain.

A radiographic study of the GB suggested that CM is characterised by the shortening of the basicranium and supraoccipital bones with compensatory lengthening of the parietal bone[15]. Morphometric analysis of MRI of the hindbrain of dogs with CM and SM has been successfully applied to a cohort of Griffons Bruxellois (GB) dogs [24] and suggested that insufficient room for the forebrain may contribute to caudal displacement and overcrowding of the hindbrain. Six traits from this study were subjected to a whole-genome association study and shown to be associated with five Canis Famililiaris Autosomes (CFAs) CFA2, CFA9, CAF12, CFA14, and CFA24. Also identified was a candidate gene, *Sall-1*, for canine CM [27]. In mice, deficiency of *Sall-1* is associated with decreased olfactory bulb size and defects in the human orthologue can be associated with skull abnormalities[28]. The olfactory bulb of brachycephalic dogs is ventrally orientated [29] and it is postulated that this may be more extreme in CM. Furthermore, these segregated traits were additive towards the severity of the CM phenotype and one trait played a protective role for GB dogs at risk for SM [30]. In the CKCS, a genome wide linkage study also identified a novel locus for SM associated with CM and a haplotype that inferred protection against SM [31].

Brachycephaly is defined as foreshortening of the facial skeleton with restricted growth of the basioccipital and basisphenoid bones manifesting as a shortening of the basicranial axis [32]. The CKCS breed description [33] indicates a greater cranial facial length than the GB, and although head conformation studies show an increased cranial index to be a risk factor for SM [34], it is not known if a more ventrally orientated olfactory bulb or rostral forebrain flattening are risk factors.

It is hypothesised that the clinical consequences of CM and SM that result from changes in brain and spinal cord conformation in the GB are the same as those in the CKCS and that segregated traits including brachycephaly are additive to the severity of the condition.

### Study Aims and Clinical Significance

The study aimed to quantify symptomatic CM, SM and associated brachycephaly in the CKCS breed by

1. mapping the hindbrain and craniocervical junction using refined morphometric techniques previously validated in the GB [24] and mixed breed [30]. These employed T1-weighted (T1w) MRI in the mid-sagittal plane.
2. quantifying brachycephalic conformation in association with CM/SM by mapping the entire brain to include rostral forebrain flattening and olfactory lobe rotation on T2-weighted (T2w) MRI in the mid-sagittal plane.

It is envisaged that the generated morphometries might increase understanding of the pathophysiology of CM/SM and assist in diagnosis. It is also hoped that identifying traits for symptomatic CM and SM could be used in conjunction with the British Veterinary Association (BVA)/Kennel Club (KC) CM/SM screening scheme and contribute towards estimated breeding values to reduce risk of pain and neurological dysfunction through selective breeding.

### Ethics Statement

This retrospective study analysed Digital Imaging and Communications in Medicine (DICOM) data obtained from dogs that underwent MRI either for diagnostic purposes for assessment of CM/SM status prior to breeding or for diagnostic investigation of neurological signs and/or pain The study was approved by the local ethics committee at the University of Surrey (reference NASAP-2015-001-SVM).

## Materials and Methods

### Study Dogs

The study dogs comprised 162 CKCS. The DICOM data was identified from two sources:-

1. 90 CKCS undergoing diagnostic investigation that included T1w imaging of the brain at the Stone Lion Veterinary Hospital (SLVH) or DICOM that has been sent to CR for the purposes of diagnostic interpretation and inclusion in the genetic study [31]. All imaging data was obtained from MRI machines which ranged from 0.2 to 3 Tesla (T) and selected because they were accompanied by DNA samples suitable for genetic studies.
2. 72 CKCS undergoing diagnostic investigations at Fitzpatrick Referrals Ltd (FR) over a two year period using a 1.5T scanner (Siemens Symphony Mastro Class, Enlargen, Germany).

CM/SM assessment was based on the BVA/KC CM/SM Health screening scheme [7] which grade both CM and SM and take account of the age of the dog at the time of screening. Since all the CKCS had CM, the dogs were divided into groups according to their SM status and if they were symptomatic or not. SM can be a progressive condition [4,35] but not always. In a study of CKCS, 43% of the dogs did not show progression of the disease [36]. Therefore in this investigation, asymptomatic dogs confirmed clear of SM at 5 years or older were selected when possible or age matched. SM is defined as a fluid filled cavity that includes or is distinct from the central canal of the spinal cord and graded according to its maximum internal diameter in a transverse plain. SM0 has no syrinx or central canal dilation. SM1 has a central canal dilatation which had an internal diameter less than 2mm. SM2 has a syrinx ≥ 2mm and only this grade was considered SM affected. Dogs screened for CM/SM prior to breeding did not have a neurological examination but all symptomatic dogs underwent a neurological examination. Clinical signs [1] included MRI to the cranial lumbar region as standard but more caudal if the dog had any clinical signs of lumbar pain.

### MRI DICOM Data

T1w sagittal images were used in the previous GB study because they provided optimum resolution for bone density. T2w sagittal images have a better resolution for distinguishing cerebrospinal fluid (CSF) and provided optimal resolution for the olfactory bulb. In this investigation the intra-reliability of measurements made on T1w and T2w DICOM for the same dog was not reliably consistent. In view of this and the fact that not all study dogs had both MRI sequences available, two parallel studies, A and B, were conducted which included the same dogs where possible.

1. T1w sagittal images investigating hindbrain and craniocervical junction abnormalities hereafter called ‘Hindbrain study’
2. T2w sagittal images investigating brachycephaly and whole brain abnormalities hereafter called ‘Whole Brain study’

The DICOM data were divided into three groups:

**Control cohort:** unaffected CKCS without SM without any clinical signs and/or behavioural signs of pain reported by their owners. These comprised SM0 and SM1 dogs that were five years or over or age matched dogs.

**CM Pain cohort:** CKCS without SM but with clinical and/or behavioural signs of neuropathic pain for example vocalisation, unwillingness to exercise and being withdrawn. These signs had been consistent over months and other sources of pain had been eliminated. They were SM0/SM1 dogs of any age.

**SM cohort**: CKCS with SM with or without clinical and/or behavioural signs of pain. These comprised SM2 dogs of any age.

Table 1 provides the ages and numbers of dogs assigned to each group. In order to limit ambiguity, the Control cohort in the hindbrain study comprised SM0 dogs only over the age of 5 years (mean age 6.2 years, median 5.4 years). The Control cohort for the whole brain study mean was age 4.4 years (median 4.9 years).

**Table 1:** Study groups with numbers and ages of CKCS in the three study cohorts.

	Hindbrain study				Whole brain study			
age	Control	CM pain	SM	Total	Control	CM pain	SM	Total
≥5years	31	16	18	**66**	7	11	7	**25**
3–4.9 years	0	4	23	**27**	7	6	7	**20**
<3yrs	0	8	30	**37**	2	8	17	**27**
**Total**	**31**	**28**	**71**	**130**	**16**	**25**	**31**	**72**

### Hindbrain Study

The study included the T1w mid-sagittal images of 130 (78 females and 52 males) CKCS. Minimum inclusion criterion was imaging of the hindbrain and cervical region. All 90 images from SLVH plus 40 T1w images from FR. The images were anonymised and all measurements were taken by author SPK, initially blinded to SM status, using a DICOM reading software package Mimics® 14.12 Materialise (15 3001 Leuven Belgium).

**Fig 1:**
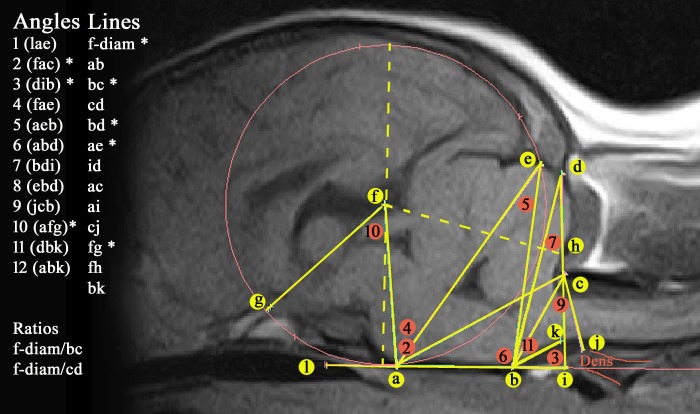
24 measurements used to map the hindbrain and craniocervical junction on T1w mid-sagittal MRIs of a CKCS without SM. Key. (a) dorsum of spheno-occipital synchondrosis. (b) basion of basioccipital bone. (c) rostral edge of the dorsal lamina of the atlas. (d) junction between the supraoccipital bone and the occipital crest. (e) most dorsal point of intersection of the cerebellum with the occipital lobe circle. (f) centre of occipital lobe circle placed on the baseline at the level of the basioccipital bone (ab) and extending to encompass the occipital lobes. Diameter of circle = f-diam. (g) point at which the optic nerve deviates into the optic canal. (h) rostral edge of supra-occipital bone. (i) intersection point with ventrally extended line dc with the caudally extended ab baseline (forms angle 3 dib). (j) most rostral aspect of the dens of the axis bone. (k) extended line from point b along the best fit line of the ventral medulla oblongata to where it changes angle to the spinal cord. (l) rostral extension of baseline abi (hence becoming baseline labi). 11 angles measured are (1) lae, (2) fac, (3) dib, (4) fae (5) aeb (6) abd (7) bdi (8) ebd (9) jcb (10) afg (11) dbk. * significant for CM in the Griffon Bruxellois [24].

Fig 1 illustrates the 24 measurements taken (13 lines and 11 angles) and used to construct a ‘grid’ that generated a craniocervical ‘map’ of each sagittal image in the study. Five lines and three angles that were significant for CM in the Griffon Bruxellois are marked with a * [24].

The measurements used in the original GB investigation were augmented to include:

1. The position of the odontoid process (dens) relative to the atlas since this was thought to impact on the degree of cranial cervical stenosis, angling of the medulla and/or obstruction of CSF channels.
2. Additional triangulation of angles arising from the basicranium to landmarks in the cranial caudal fossa to reflect any overcrowding of the cerebellum and medulla oblongata.

### Whole Brain Study

This study of 72 CKCS (39 males and 33 females) used anonymised and randomised T2w sagittal images of whole brain from FR. The minimum inclusion criteria was imaging of the entire brain parenchyma and cervical region. In order to ensure consistency with the hindbrain study, 32 dogs that had both T1w and T2w were included (i.e. overlapped), together with 11 ‘hindbrain’ measurements. These comprised: angles 2,3,7,9 and 10, lines ab, bc, ac, ai, and bk and the diameter of ‘best fit’ occipital circle with centre at f (f-diam). All these and the additional measurements i- iii listed below were recorded by author SPK using the DICOM reading software package Mimics^®^.

**Fig 2:**
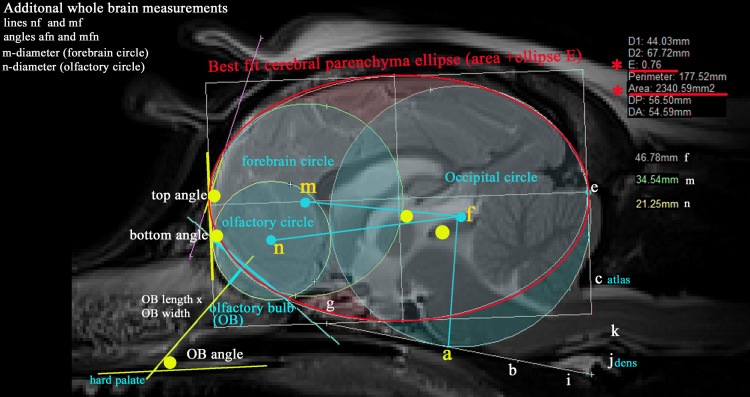
Additional morphometric measurements taken of the T2w mid-sagittal brain MRI of a CKCS with CM pain. Key For identity of points a-l see Fig 1. Three ‘best fit’ circles (coloured aqua) and an ellipse (coloured red) that follow the outline shape of the neural parenchyma as closely as possible. Occipital circle (centre f)–as for hindbrain study. Size is determined by the shape of the occipital lobe extending rostroventrally to the baseline labi (basioccipital bone). i Forebrain circle (centre m)—most rostral portion of the forebrain dorsal to the cribriform plate of the ethmoid bone. ii Olfactory circle (centre n)—size is determined by the shape of olfactory bulbs extending beyond the pre-frontal cortex in the mid-sagittal image. iii Cerebral parenchyma ellipse which encompasses the caudal edge of the occipital and rostral edge of the forebrain circle (i.e. the cerebral parenchyma but not including the entire cerebellum or brainstem), its area (mm^2^) together with its ellipticity ‘E’ (defined as a mathematical relationship between of the largest radius to the smallest radius in the ellipse). Both calculated by Mimics Materialise® software programme. (smaller E values = more spherical, larger E values more elliptical). iv Associated lines (coloured aqua) comprising mf, nf, The olfactory bulb (OB) length and height (product represented. v Five angles (coloured yellow): • top angle—angulation between the frontal and parietal lobes. • bottom angle -angulation between the dorsal OB and the frontal lobe. • OB angle—angulation between the OB and hard palate. • mfn and nfg.

Additional measurements illustrated in Fig 2 included:-

i Forebrain circle (diameter m) and distance/line mf
ii Olfactory circle (diameter n), the distances/line nf and angles mfn and nfa
iii Total brain area and its ellipticity (E). Ellipticity is defined as a mathematical relationship between of the largest radius to the smallest radius in the ellipse and measures how ovoid the shape is. Both E and brain area were calculated automatically by the Mimics® software programme.

A further 6 measurements were recorded by author CC viewed in eFILM ™ 18.

iv two measurements that represented flattening of the rostral forebrain
v olfactory bulb size (length and width)
vi the angulation of the olfactory bulb with the hard palate

### Morphometric Mapping and ‘Morphing’ Technique

Multiple measurements were taken which used both bone and tissue landmarks to explore the brain and craniocervical junction. Because the lines and angles are interrelated, any deviations from the ‘normal’ juxtaposition of hindbrain, spinal cord and skull could enhance understanding of the pathogenesis of CM and SM. To take account of differences in natural anatomical variations in the CKCS, particularly size of head, two trait ratios were included in the analysis. These were related to the height of the cranial fossa to the representative distance across the foramen magnum (f-diameter/line bc) and the height of the supraoccipital bone (f-diameter/line cd). Advanced statistical analysis has the potential to ascertain those traits best suited to identify the abnormalities associated with CM/SM.

The morphometries, as with the GB study, are underpinned by a ‘best fit’ occipital circle f. This follows, as closely as possible, the contour the occipital lobes dorsal to the cerebellum and extends caudally and ventrally to the level of the skull base (i.e. basioccipital bone). The diameter of the circle provides a linear value of the approximate height of the caudal cranial fossa parenchyma, while the circle radius provides a proportional distance to assess the juxtaposition of anatomical features such as the interthalamic adhesion, sella turcia, etc. Moreover, the circle provides an important means of standardising the morphometric traits between various sizes of dog.

Adobe Photoshop 34564^®^ (http://www.adobe.com) was used for graphic analysis and to provide images for morphing. Jpegs of the images generated by Mimics^®^ were imported into Photoshop®. By applying the ‘maintain fixed ratio’ tool of the software, the images were resized so that the occipital circle was the same size in all exemplar images and the skull baseline labi was rotated to the horizontal plane and aligned to facilitate comparison of skull angulations. These images were used to make a photo morphing movie using Abrosoft Fantamorph^®^ 5.4.5 software (http://www.abrosoft.com).

### Statistical Analysis

IBM SPSS for Windows^®^ version 22 was used to calculate measurement reliability (Intraclass Correlation Coefficient (ICC) model) and to analyze variables in both the hindbrain and whole brain cohorts independently but identically. Analysis of Variance (ANOVA), with a Bonferroni correction for multiple testing was used to analyze the traits for total cohorts. The independent sample t-test with Levene's test for equality of variance was used for differences between all three possible paired subgroup combinations (Control, CM pain and SM). Descriptive statistics (box plots) were generated for selected significant variables. P-values (bold) were considered significant: < 0.05 for ANOVA and with Bonferroni correction, < 0.017 for the t-test. Since segregated traits associated with CM and SM have been shown to be additive to the severity, Discriminant Function Analysis (DA) was applied to the data in order to examine the relationships between the significant variables in more depth. DA is helpful to identify the most important phenotypic trait variables that distinguish between each group. In such an analysis the selected traits are evaluated by using cross-validation to avoid data bias and to confirm the prediction model. DA takes account of any correlations between variables and how reliable these are for predicting the group to which each dog had been assigned.

### Reliability Analysis

Measurements of 2 lines, 2 angles and 1 circle from ten dogs were obtained by author SPK and these measurements repeated one year later in order to assess intra-observer reliability. Ten dogs were also measured independently by authors CC and CR to assess inter-observer reliability. After these measurements were obtained, CC then made an independent second measurement of 10 cases to ensure high intra-observer reliability.

## Results

Inter-rater reliability was found to be very satisfactory with all ICC values in excess of 0.75. Average ICC value 0.86 for author SPK indicated a relatively high consistency maintained between the previous GB study[24] and the CKCS study. Intra-observer results for author CC all exceeded 0.96 and 95% confidence intervals were considered narrow. Overall, 50 measurements, comprising 24 hindbrain (S1 Table) and 26 whole brain (S2 Table), were made to quantify the phenotypic differences CKCS with and without SM and with CM pain.

### Hindbrain Study

In this study cohort 61 of 130 dogs (46.9%) had insufficient imaging of the forebrain prohibiting measurements of line fg and angle 10 (afg) (called angle 5 in GB study). Statistical analysis of the total group employing ANOVA identified 14 significant variables and differentiated by the three possible paired subgroup combinations (independent t-test). Table 2 tabulates these results. t-test results with p-values between 0.05 and 0.017 are also reported. The five traits associated with five CFA in previous GB studies–f-diameter, lines bc, ae and angles 2, 3 and 10 were also significant in this study.

**Table 2:** Significant traits identified in four analyses of the hindbrain study using ANOVA (1) and independent sample t-tests (2–4).

Group	1.Total cohort n = 130		2.control v SM n = 99[28,71]		3.control v CM pain n = 59[28,31]		4. CM pain v SM n = 102[31,71]	
variables	F	p-value	t	p-value	t	p-value	t	p-value
*L*3* (dib)	13.53	**<0.001**	-5.34	**<0.001**			-2.65	**0.011**
***L*5(aeb)**	13.50	**0.007**		** **	3.35	**0.001**		** **
***L*7(bdi)**	14.85	**<0.001**	4.81	**<0.001**			4.02	**<0.001**
***L*9 (jcb)**	14.80	**<0.001**			-2.23	0.030	4.59	**<0.001**
*L*10* (afg)	3.88	**0.024**					2.74	**0.008**
***L*11(ebk)**	4.30	**0.016**	2.82	**0.006**	2.26	0.027		
line f-d*	6.30	**0.002**	-4.04	**<0.001**				
**line ab**	4.10	**0.019**			2.41	0.019	-2.70	**0.009**
line bc*	6.70	**0.002**					3.68	**<0.001**
line ae*	4.90	**0.009**	-4.06	**<0.001**				
**line ac**	4.30	**0.015**	2.51	**0.014**			2.30	0.024
**line ai**	10.70	**<0.001**	4.62	**<0.001**			2.91	**.004**
**line bk**	9.50	**<0.001**	3.16	**0.002**			4.35	**<0.001**
**ratio f-d/bc**	12.40	**<0.001**	-3.66	**0.001**			-4.34	**<0.001**

*trait significant in previous GB studies *L* = angle

F: F-test from one factor ANOVA

t: t-test statistic from independent sample t-test.

### Whole Brain Study

Table 3 details nine significant traits (in bold) identified in the 72 whole brain group with p< 0.05 for ANOVA and with Bonferroni correction, < 0.017 for the t-test. Angle 2, which was uniquely significant for CM but not SM in the GB study [24,27], was significant in the total group and in combinations involving the control cohort.

**Table 3:** Significant traits identified in four analyses: 1. Total group using ANOVA, 2–4 paired cohorts using independent sample t-tests.

Group	1.Total group n = 72		2.Control v SM n = 47		3.Control v CM pain n = 41		4. pain v SM n = 56	
variables	F	p-value	t	p-value	t	p-value	t	p-value
***L*2^ (fac)**	5.93	**0.004**	2.25	**0.030**	3.41	**0.002**		** **
*L*3* (dib)	4.39	**0.016**	-2.90	**0.007**		** **		** **
***L*7 (bdi)**	4.57	**0.014**	2.95	**0.013**		** **		** **
*L*10* (afg)	4.86	**0.011**	2.79	**0.008**			-2.55	**0.014**
**line bk**	4.10	**0.021**		** **	-3.28	**0.003**		** **
**line ai**	5.20	**0.008**	3.22	**0.002**		** **		** **
**Ellipticity**	7.27	**0.010**	3.72	**0.001**	2.73	**0.010**		** **
**bottom angle**	3.47	**0.037**				** **	2.82	**0.007**
**OB angle**	3.69	**0.030**			-3.15	**0.003**		

*trait significant in previous GB studies

*L* = angle

OB = olfactory bulb

F: F-test from one factor ANOVA significance

t: t-test statistic from independent sample t-test.

### Statistical Analysis Using Discriminant Analysis

**Fig 3:**
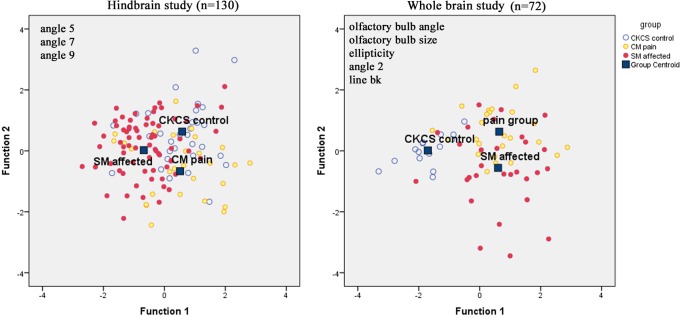
Canonical Discriminate functions of CKCS with and without CM pain and SM.Hindbrain study. Average 64.6% correctly classified with SM affected highest separation of 78.9%. Function 1 increases with larger angles 5, 7 and 9 and Function 2 increased with larger angles 5 and 7 but smaller angle 9. Thus, SM cohort has smaller angles 5, 7 and 9 than other groups because the centroid is furthest on the left. The CM pain cohort on average has larger angles 7 and 9. The Control cohort has highest centroid in Function 2 hence they would, on average, they have larger angles 5 and 7 and smaller angle 9. **Whole brain study**: Average 72.5% of the group was correctly classified with 93.3% of Control cohort. Function 1 the combined effect of decreases with of ellipticity and angle 2 (fac) but increases with lines bk, the n-diameter and the olfactory bulb angle. Function 2 increases with line bk, ellipticity and olfactory bulb and decreases with angle 2 and the n-diameter. It follows that the group has the centroid on the left side and has a more elliptic brain, wider angle 2 but smaller olfactory bulb angle (i.e. the olfactory bulb is not so rotated).

Discriminant Analysis is a method used in statistics to find linear combination of features that separates two or more groups which all have continuous variables. It proved useful in identifying a total of eight significant traits which best separated the Control cohorts from CM pain and SM cohorts. The DA canonical coefficients for each group (Table 4) are plotted in the conventional manner in Fig 3. Function F is the ratio of between group variations to within group variation with higher values indicating the likelihood of a group effect. In the hindbrain study, the angles 5, 7, and 9 were identified as most significant in DA. After cross-validation for the whole group, an average of 64.6% was correctly classified (SM 78.9%; 45.2% Control; 50.0% CM pain). In the whole brain study, five traits (line bk and n-diameter, Angle 2(fac), Ellipticity and OB angle) were all identified and on average 72.5% of the group were correctly classified (93.3% Control; 68% CM Pain; 65.5% SM affected).

**Table 4:** Canonical Discriminant Function Coefficients.

Hindbrain study			Whole brain study		
Function	1	2	Function	1	2
***L*5 (aeb)**	0.089	0.283	**bk**	0.188	0.351
***L*7(bdi)**	0.143	0.03	***L*2 (fac)**	-0.133	-0.047
***L*9 (jcb)**	0.057	-0.068	**n- diameter**	0.226	-0.378
**(Constant)**	-8.437	-4.924	**Ellipticity**	-0.363	0.219
			**Olfactory Bulb Angle**	0.078	0.038
			**(Constant)**	23.538	-10.499

*L* = angle

**Fig 4:**
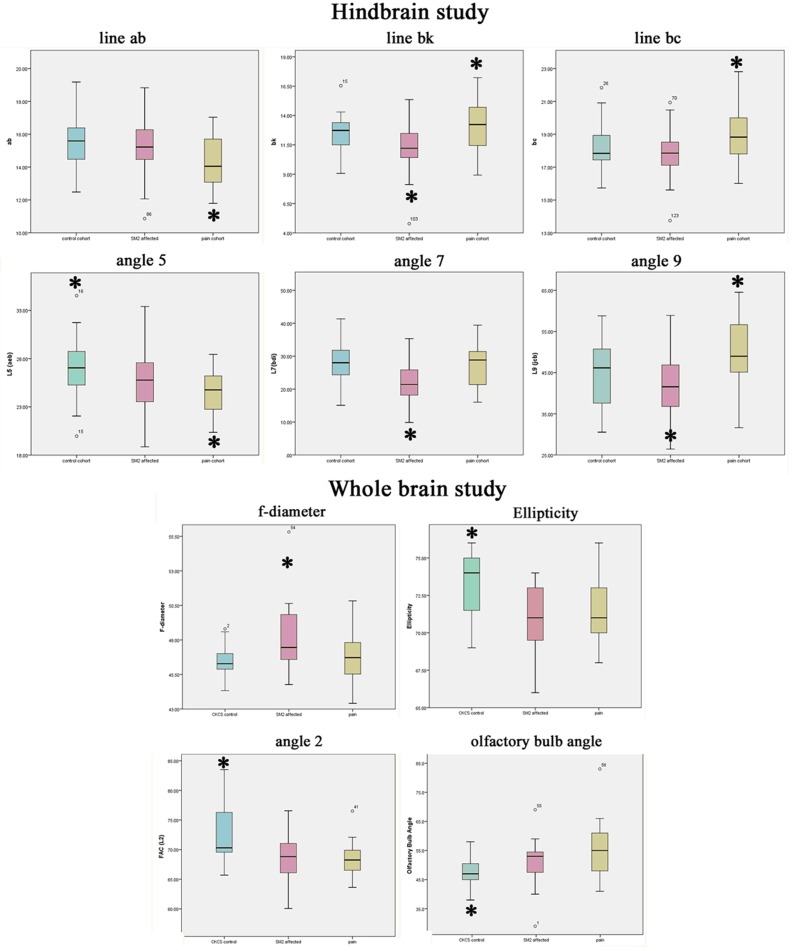
Descriptive boxplots of key significant traits for the 3 study cohorts; Control, CM pain and SM. Colour key: blue = Control; pink = SM; yellow = cm pain * highlights significant trait referenced in text summary.

Fig 4 provides descriptive boxplots of a selection of variants used in the text which distinguish significant differences between the three groups.

### Summary Group Findings

**Control cohort**; has the most ovoid (least spherical) shaped elliptical brain with a tendency towards a wider angle 2 (p = 0.004) and angle 5 (p = 0.007) with least ventral rotation of the olfactory bulb (p = 0.030) i.e. it is the least brachycephalic group compared to the others.

**CM pain cohort**; has a short basicranium (line ab) with a resultant compensatory increased cranial height (small angle 7) and increased brachycephaly with olfactory bulb more ventrally rotated (p = 0.003) and rostral forebrain flattening (p = 0.007) compared to Control CKCS. However, in comparison with SM dogs, the cohort has a longer line bc and a wider angle 9 increases the volume of the caudal fossa, which may lessen obstruction to CSF flow and the risk of developing SM.

**SM cohort**; has a tendency towards a bigger f-diameter (p = 0.002) i.e. greater compensation to rostral caudal shortening, smaller angles 7 and 9 and shorter line bk (p <0.001) at the craniocervical junction compared to other groups. The additive effects of other traits give two phenotypic variables predisposing to SM. These are:

1. Reduced supra and basioccipital bone lengths with an increased proximity of both the atlas and the dens. All these anomalies reduce the volume of the caudal fossa (wide angle 3, short line bc);
2. More brachycephalic (smaller angles 2, 5 and 10) with compensatory cranial height (f-diameter), with the hindbrain being invaginated into the cranial fossa and the craniocervical vertebrae invaginated towards the foramen magnum.

**Fig 5:**
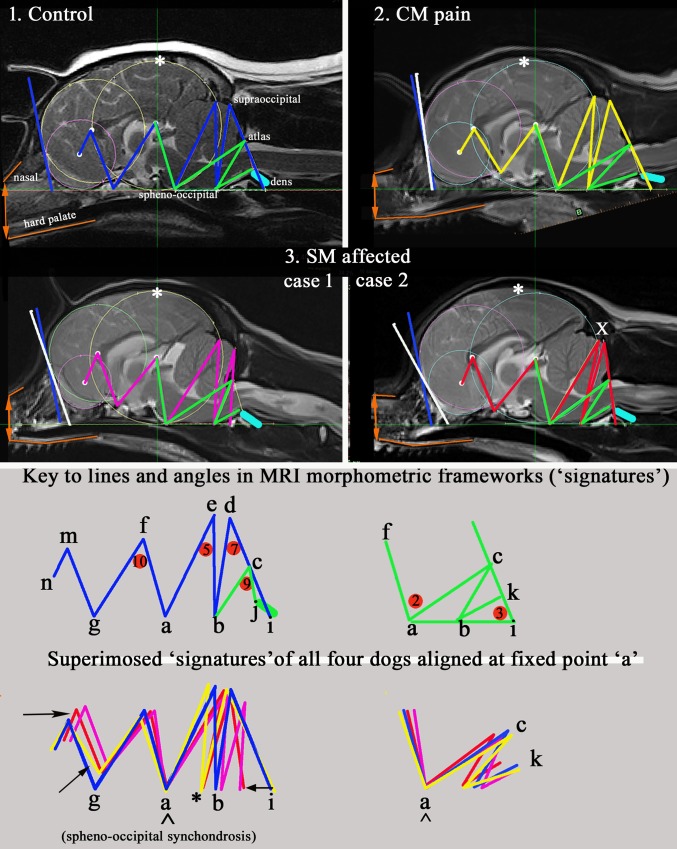
Four mid-sagittal T2w whole brain MRI exemplars of cohorts Control, CM pain and two conformation cases of SM. The occipital circle has been standardised in all the images and the baseline labi aligned to facilitate comparison. Colour codes for morphometric ‘signatures’: Blue = Control; Yellow = CM pain; Red/Crimson = SM affected (two cases). Green: all groups = angles 2 and 3 and lines bc and bk. Superimposed- CM pain (yellow) has most extreme range. White: *greatest parenchyma height from skull baseline and is rostral to occipital circle in CM pain and SM affected case 2. x caudal displacement of occipital lobe. white/blue bar drawn at most rostral point of forebrain and olfactory circles indicates angulation of forebrain flattening. The blue bar (Control dog) has been superimposed on the three other group dogs for comparison. CM pain has greatest rostral forebrain flattening; The SM case 2 has the greatest olfactory lobe deviation.Orange: brachycephaly- lines mark the position and relationship of the upper nasal bone and the hard palate. SM case 2 has the greatest brachycephaly with angulation at the nasion and the lower palatine/incisive bones. Aqua: dens. This lies closest to the basioccipital in SM case 1. The different angle of dens in CM pain dog was found to be significant for the study. Black: arrows suggest displacement resulting from craniosynostosis. * indicates deviation (shortening) of occipital bone in CM pain and SM case 2.

Fig 5 should be viewed with its accompanying morphing movie (S1 Movie). They illustrate four exemplars of the key findings for the three study cohorts: Control, CM pain and two conformation variations associated with SM, Cases 1 and 2. The movie in particular is a powerful illustration of the impact of progressive brachycephaly and airorhynchy when combined with the incongruities of the reduced caudal fossa and craniocervical junction. Fig 3 also exposes the relationship of the nasal bone and hard palate (coloured orange) with the cranium and the angulation of the nasion (stop) which increases with brachycephaly.

## Discussion

### Multifactorial Nature of CM/SM

The morphometric mapping of the CKCS in both the hind and forebrain studies revealed the complex nature of CM/SM and its relationship with aspects of brachycephalic conformation[16,34]. Genetic mapping of head conformation associated with brachycephaly has identified several candidate genes and supports its multifactorial origins [37,38]. Variations in size and shape of the skull known to exist in CKCS breed, the late onset nature of SM, the spectrum of clinical signs for CM/SM and difficulty of identifying behavioural pain all add to the complex nature of the condition. For these reasons, the characterisation of symptomatic CM and SM affectedness focuses on the anatomical variations associated with these morbidities rather than the traits associated with ‘normal’ CKCS conformation which can be quite diverse. The use of the ‘best fit ‘occipital circle with the f-diameter and its ratio with the traits representing the size of the foramen magnum (line bc) and supra-occipital bone (line cd) have been key features in the characterisation. The early closure of the of the sphenoid occipital synchondrosis in the CKCS [23] makes the landmark ‘point a’ (Fig 3) a useful anchor point to superimpose the exemplar dogs’ morphometric ‘signatures’ in order to compare them.

### Conformation similarities with griffon bruxellois

The five significant variables for SM (f-diameter, lines bc and ae and angles 3 and 10) were identified in both GB and CKCS suggesting a common aetiology. Furthermore, a smaller angle 2 (fac) which was significant for CM but not SM in the GB study [24] and a wider angle 2 was shown to have a protective role against SM in a mixed breed family [30]. In this study, angle 2 was significant in the forebrain study (control v CM pain and SM). Genetic studies [27] have confirmed that the increased f-diameter (significant locus CFA2 and the CM candidate *Sall-1* gene) and increased length of lines ae and fg (significant locus CFA14) reflect the increased height of the cranium and rostral caudal fossa. It is hoped that the current CKCS genetic studies will shed some light on the aetiology of both CM and SM [31,39].

### Brachycephaly and airorhynchy

Although the CKCS is recognised as having a brachycephalic skull [40], facial length is very varied in the breed, with the muzzle becoming fashionably shorter and more dorsally rotated (airorhynchy) in the last decade [34,40]. It is entirely credible that craniofacial conformation makes a significant contribution to CM and risk to SM as it does in the GB breed [15,24,41]. Thus, CM is not just a reduction in the cranial base and caudal fossa. The ‘ellipticity’ of the brain provides a quantitative value to compare the natural oval shape of the Control cohort to the more global brachycephalic CM pain and two SM cases. The reduced size and rotation of the olfactory bulb, together with the clival angle (cranial base angulation between the ethmoidal plane and the clival plane [42]), is associated with a shortened muzzle and increased stop and a ‘face’ that tilts up like a human. This is illustrated in Fig 3 which have the nasal and palate bones highlighted as orange lines. The morphing movie (S1 Movie) highlights the dynamic changes of the skull conformation and brain parenchyma associated with progressive brachycephaly and airorhynchy, shortening of the basicranium and supraoccipital bones and the proximity and angulation of the atlas and dens.

A recent study of suture closure and skull morphology in dogs[43] investigates the prebasial angles (angle between the hard palate and the cranial base of skull). These suggest that as the phenotype morphs from normal (Control) to CM pain and then to SM affected there is increasing airorhynchy. This is recognised as greater retroflexion of the facial skeleton on the cranial base in the most extreme case (SM case 2) which also has the greatest olfactory bulb rotation and ‘stop’ (nasion). The olfactory bulb links directly to the subarachnoid space via the cribriform plate of the ethmoid bone[44]. Any reduction in its size would impact on the absorption through the choroid membranes [44] and influence CSF dynamics. In humans the clivus-supraoccipital angle has been used to predict the size of fetal posterior fossa and diagnose CM11 malformation [45].

### Craniocervical junction conformation impact on CSF flow dynamics

Angle 3 (dib) plays a major role in the morphometric analysis in the hindbrain study since it links both the supraoccipital and atlas bones with the basicranium. It also contributes to angle 7 and linked to lines ac, bc and bd. These quantify areas within the caudal fossa. The alignment of Angle 3 is independent of the dens but the relationship between its proximity and angulation can be visualised with morphometric mapping and is particularly significant with respect to SM and Control (Table 2, p <0.001). Conversely, line bc measures the distance from the occiput to the atlas across the foramen magnum and the increased length in the CM pain cohort is an important distinction between this group and the SM cohort (Table 2, p **<**0.001).

The craniocervical junction is the bony gateway linking the subarachnoid spaces of the brain and the spinal cord and altered conformations affects the CSF dynamics. A current theory to the development of syringes in the spinal cord is the imbalance of venous volume and timing with the filling and draining mechanisms within the subarachnoid spaces of the brain and spinal cord. Impedance studies have been made in humans [46,47] and morphometric measurements of the human clival angle have demonstrated that a wider angle is correlated with a decrease in the width of the foramen magnum, but not the height [42]. These variants are associated with fetal ventriculomegaly [45].

### Clinical Application

Quantitative mapping may provide an additional diagnostic tool in distinguishing CM pain which has previously been difficult. Since SM dogs can have added CM pain, distinguishing between CM and SM associated pain may also be possible. This study provides objective values that can estimate the additive effects that result in cranial overcrowding. Currently the BVA/KC Health CM/SM screening scheme uses deformation of the cerebellum as the criteria for CM but this study shows that account should be taken of brachycephaly and other brain and craniocervical junction conformational changes and possibly the degree of airorhynchy. There is a need to make predictive risk assessment for both CM and SM to assist breeders with their breeding decisions. However, the number of complex measurements made in this investigation would be impractical in any screening scheme. It is proposed that the development of a software package might allow digital qualification of these traits to give the dogs an overall ‘score’ that could be correlated to phenotype to predict disease susceptibility.

### Limitations of the Study

Although allowance was made for the variable quality images by selecting the most obvious landmarks, the initial placement of a single line on the curvature the cranial base was sometimes difficult. In order to overcome this, a large number of inter-related measurements were made to mitigate any deviation. Since robust phenotyping is paramount for intended genetic studies, screenshots were also made of each dogs ‘signature’ for reference so they could be checked visually for consistency.

The study was somewhat limited by the number of entire brain MRI images available for the Control and CM pain cohort. 35% of the latter group were less than 5 years of age and may go on to develop SM, but these dogs were age matched. Screening asymptomatic dogs over 5 years is not common and the BVA/KC/ CM/SM Health breeding scheme [7] does not require MRI of the whole brain. Furthermore, it relies on the asymptomatic assessment by the owner and some clinical and/or behavioural signs of pain may not be recognised. Despite ruling out other clinical causes in the CM pain cohort, the interpretation of the behavioural signs of pain remains subjective and intermittent signs may be overlooked and others over-interpreted.

## Conclusion

Morphometric mapping using a triangulation of lines, angles and circles is useful for defining SM and CM pain phenotypes. The results confirm that it is essential to consider the whole brain in the characterisation of CM which takes account of the brachycephaly and its additive effect on CM/SM. Through the standardisation of the ‘best fit’ circles and ellipse, it is possible to quantify differences in conformations associated with brachycephaly and the proximity of the cervical vertebrae to the skull that result in CM pain and SM. Linking the angles and lines to create a unique ‘signature ‘for each dog enables comparisons to be made relative to size and altered position of anatomical features. The Control cohort had the most natural, wolf-like, skull conformation in terms of ellipticity. The CM pain cohort was characterised by increased brachycephaly with greatest rostral forebrain flattening, shortest basicranium and compensatory cranial height. However, in this cohort, an increased distance between the occiput and atlas provided fewer impediments to CSF dynamics at the foramen magnum and reduced the risk for SM. The SM cohort exhibited two conformation anomalies. One phenotype variation was influenced by incongruities at the craniocervical junction and increased proximity of the dens producing a ‘concertina’ type flexure with medullary elevation. The other phenotypic variation was influenced by increased brachycephaly resulted in a ‘concertina’ type flexure similar to the CM pain cohort. However, both SM variations were characterised by an apparent reduction in caudal fossa volume which compromised the CSF dynamics in the spinal cord.

The possibility of quantifying the phenotype with a digital morphometric mapping tool is discussed. It might identify dogs at risk of SM and CM pain to improve diagnosis and make available a means for screening breeding dogs and provide estimated breeding values.

## Supporting Information

S1 Table Morphometric measurements made of the hindbrain study cohort. (XLSX)

S2 Table Morphometric measurements made of the whole brain study cohort. (XLSX)

S1 Movie Morphing morphometric ‘signatures’ of four exemplar sagittal whole brain T2w MRI provided in Fig 3; Control, CM pain and two conformation cases of the SM cohort. The movie highlights the dynamic changes of the skull conformation and brain parenchyma associated with progressive brachycephaly and airorhynchy, shortening of the basicranium and supraoccipital bones and the proximity and angulation of the atlas and dens. Using a ‘fixed ratio’ image tool, the occipital circle of the four exemplar MRI images has been standardised and the baseline abi aligned, the movie graphically illustrates the concertina flexure of the dog morphometric signatures with changed CM and SM status. As the video morphs from the control dog to one with CM pain that the nasal bone and hard palate become closer so that the hard palate becomes more horizontal, the nasal cavity and frontal cavity reduce in volume and the rostral forebrain is flattened. As the model progresses into SM case 1 the nasal and rostral forebrain changes become more extreme. In addition to the forebrain changes, the hindbrain is pushed caudally and the craniocervical junction kinks as a consequence of the cervical vertebral being closer to the skull with flattening of the supraoccipital bone. Consequently there is a “concertina” flexure of the brain with a compensatory increase in height of the cranial fossa (asterisk). In SM case 2 the concertina flexure is more extreme caudally (X) with the cerebellum invaginated under the occipital lobe and the olfactory bulbs are much reduced in size and ventrally displaced.
(ZIP)
